# Employment History and Later-Life Health

**DOI:** 10.1093/geroni/igaf122.187

**Published:** 2025-12-31

**Authors:** Wenxuan Huang

## Abstract

Employment provides essential financial resources, institutional protection, and social networks to workers, all of which serve as key social determinants of health throughout the life course. While the link between employment and health is well-documented, limited research has taken a holistic approach to examine how employment histories shape later-life health. Recent advances in longitudinal research methods and the growing availability of life history data have enabled new efforts to address these gaps. This symposium brings together four studies investigating the impact of employment history on various health outcomes in later life across different social contexts. Using data from Health Retirement Study, Han and Dr. Moen’s apply advanced causal estimation models to assess how workforce participation in the 50s contributes to cognitive health in later life in the U.S. context, highlighting both time-varying effects and subgroup heterogeneity. Dr. Fu et al.’s paper explores how the life-course patterns of work history shape the frailty trajectories among Chinese adults aged 60 and older, emphasizing the role of cumulative disadvantage in work on frailty. Dr. Han’s study examines the temporal pattern of employment history using sequence analysis approach and assesses its association with late-life functional limitations, revealing the urban-rural divide in employment patterns and its health consequences in China. Dr. Huang’s paper combines sequence analysis and decomposition method to understand how typical employment patterns explains the midlife health disparities between high school graduates and GED recipients. By conducting life-course-sensitive analysis, these studies collectively highlight how unequal employment histories drive later-life heath disparities.

